# Laser capture microdissection of gonads from juvenile zebrafish

**DOI:** 10.1186/1477-7827-7-97

**Published:** 2009-09-14

**Authors:** Anne Jørgensen, John E Nielsen, Jane E Morthorst, Poul Bjerregaard, Henrik Leffers

**Affiliations:** 1 Department of Science, Systems and Models, Roskilde University, Universitetsvej 1, DK-4000 Roskilde, Denmark; 2 Institute of Biology, University of Southern Denmark, Campusvej 55, DK-5230 Odense M, Denmark; 3 University Department of Growth and Reproduction, Rigshospitalet, Blegdamsvej 9, 2100 Copenhagen ∅, Denmark

## Abstract

**Background:**

Investigating gonadal gene expression is important in attempting to elucidate the molecular mechanism of sex determination and differentiation in the model species zebrafish. However, the small size of juvenile zebrafish and correspondingly their gonads complicates this type of investigation. Furthermore, the lack of a genetic sex marker in juvenile zebrafish prevents pooling gonads from several individuals. The aim of this study was to establish a method to isolate the gonads from individual juvenile zebrafish allowing future investigations of gonadal gene expression during sex determination and differentiation.

**Methods:**

The laser capture microdissection technique enables isolation of specific cells and tissues and thereby removes the noise of gene expression from other cells or tissues in the gene expression profile. A protocol developed for laser microdissection of human gonocytes was adjusted and optimised to isolate juvenile zebrafish gonads.

**Results:**

The juvenile zebrafish gonad is not morphologically distinguishable when using dehydrated cryosections on membrane slides and a specific staining method is necessary to identify the gonads. The protocol setup in this study allows staining, identification, isolation and subsequent RNA purification and amplification of gonads from individual juvenile zebrafish thereby enabling gonadal gene expression profiling.

**Conclusion:**

The study presents a protocol for isolation of individual juvenile zebrafish gonads, which will enable future investigations of gonadal gene expression during the critical period of sex differentiation. Furthermore, the presented staining method is applicable to other species as it is directed towards alkaline phosphatase that is expressed in gonocytes and embryonic stem cells, which is conserved among vertebrate species.

## Background

Zebrafish is used extensively as a model species for studies on vertebrate development and for assessing effects of endocrine disrupting chemicals on reproduction. Despite this, the molecular mechanisms controlling zebrafish sex determination and gonadal differentiation are poorly understood [1-3] In order to determine gene expression during the early gonadal development it is necessary to investigate the first 20 days post hatch (dph) [4]. However, due to the small size of the juvenile zebrafish it is difficult if not impossible to dissect gonads from individual fish and as gonads from different individuals cannot be pooled due to the lack of an early sex marker, an alternative strategy is needed. Therefore, the aim of the present study was to establish a method that allows identification, isolation and subsequent RNA purification of the gonads from individual juvenile zebrafish thereby allowing investigation of gene expression during the expected time of sex determination and differentiation.

Microdissection is a powerful tool to isolate specific cells or tissues and thereby ensure a specific gene expression profile without noise from other cells or tissues. When cryosections are used it is possible to avoid total degradation of RNA, however, when microdissecting tissue from frozen and dehydrated juvenile zebrafish, the morphology is impaired and it is difficult to distinguish between the different tissues. The widely used haematoxylin eosin (HE) staining is not sufficient for identification of the juvenile zebrafish gonads for microdissection and therefore a specific staining protocol is necessary. Previous studies have shown that fetal germ cells (gonocytes) have embryonic stem cell like properties including expression of alkaline phosphatase [5-10] Alkaline phosphatase can be detected by staining with Nitro-Blue tetrazolium chloride and 5-Bromo-4-chloro-3-indolyl phosphate (NBT BCIP) and this has previously been applied for identification of human gonocytes followed by microdissection, RNA purification and linear amplification [11].

## Methods

### Animals

Juvenile zebrafish originated from a brood population of fish. In the evening breeding boxes were placed in an aquarium with parent fish and eggs were collected the following morning. Non-fertilised eggs were removed while the fertilised eggs were placed in 900 ml glass beakers and kept at 26 ± 1°C and a light-dark period of 14:10 h. In the interval 3-22 dph the larvae were fed two times daily with powdered dry food (Sera Micron) and one time daily with newly hatched artemia sp. nauplii (Intér Ryba GmbH, Germany). At 5, 10, 15 and 20 dph zebrafish were frozen individually in liquid nitrogen and stored at -80°C until cryosectioning and NBT BCIP staining. Zebrafish used for in situ hybridisation (5, 10, 15 and 20 dph) were fixed in Stieves fixative (solution I: 90 g HgCl_2 _in 1.5 L H_2_O; Solution II: 400 g formaldehyde and 80 g glacial acetic acid in 1 L H_2_O; just before use mix 38 ml Solution I and 12 ml Solution II) at room temperature for 24 hr (fixatives from VWR, Copenhagen, Denmark).

### NBT BCIP staining for alkaline phosphatase in gonads

Zebrafish were embedded in Tissue-Tech Optimal Cutting Temperature (OCT) compound (Sakura Fintek Europe, Zoeterwonde, NL), rapidly frozen in isopentan on dryice and stored at -80°C until further analysis. The frozen tissue was cut in 20 μm serial sections on a Cryostat and either mounted on RNase free membrane slides (Molecular Machines & Industries, Glatbrugg, Switzerland) for microdissection or on Superfrost slides for HE staining. The membrane slides were fixed immediately in 75% ethanol for 10 min. at room temperature, stored in 100% ethanol in -80°C and was then stained with NBT BCIP as described previously [11]. In short, membrane slides were placed: 10 sec in incubation buffer, 90-120 sec. in NBT BCIP solution [262.5 μg/mL *p*-Nitro-Blue tetrazolium chloride; 225 μg/mL 5-Bromo-4-chloro-3-indolyl phosphate dipotassium salt (both from Sigma-Aldrich, St Louis, MO, USA); dissolved in 70% and 100% dimethyl formamide, respectively], 10 sec in DEPC water, followed by dehydration in ethanol (10 sec 62% ethanol, 2× 10 sec 96% ethanol and 2× 10 sec 100% ethanol). The stained germ cells and the surrounding area corresponding to the gonads were dissected using Olympus SmartCut microdissection system according to manufacturers instructions. Lysis buffer from RNAqueous-Micro Kit (Ambion, Austin, TX, USA) was added to the microdissected tissue as fast as possible and samples were stored at -80°C until further analysis. RNA was purified using RNAqueous-Micro Kit according to the manufacturers instructions for microdissected tissue. The quality of the RNA was analysed using Agilent Bioanalyser 2100 and Agilent RNA 6000 Pico Kit (Agilent Technologies, Santa Clara, CA, USA). The RNA was amplified using MessageAmpII™ II aRNA Amplification Kit in two rounds. After both rounds of amplification, samples were quantified using a NanoDrop ND-1000 spectrophotometer (NanoDrop Technologies, Wilmington, DE, USA).

### Reverse transcription-polymerase chain reaction

cDNA synthesis was performed using 0.5 μg amplified RNA and 50 ng/μl random hexamer primers in a final volume of 20 μl. cDNA control was performed without RNA. Reverse transcriptase polymerase chain reaction (RT-PCR) using 1 μl cDNA and gene specific primers placed just upstream of the polyA site was performed in (final concentrations): 10 mM Tris-HCl, pH 8.3; 50 mM KCl; 1.8 mM MgCl_2_; 0.1% Triton X-100; 0.0005% gelatine; 250 μM dNTP and 1 pmol/μl primer. Specific primers targeting *vasa *(fwd: CATCGCATAGGAAGAACTGGA; rev: GGCTCATCGCTCTTGAAGGAT) and *β-actin *(fwd: AGTGCGACGTGGACATCCGTA; rev: GCACTTCCTGTGGACGATGGA) were designed to span intron-exon boundaries. Cycle conditions were: one cycle of 2 min at 95°C; 40 cycles of 30 sec at 95°C, 1 min at 62°C, 1 min at 72°C and finally one cycle of 5 min at 72°C. PCR products were run on 1% agarose gels and visualized by ethidium bromide staining. Bands from each primer combination were excised and sequenced for verification (DNA Technology, Aarhus, Denmark).

### Preparation of biotin labelled probe for ISH

Probes for ISH were prepared by RT-PCR amplification of *vasa *transcripts and reamplification of PCR fragments using nested primers specific to the fragments with an added T3-promotor sequence in combination with the T7-extended downstream primer (**AATTAACCCTCACTAAAGGG**CTGTGGGAACACC and **TAATACGACTCACTATAGGG**CCCTTCCGCGGAGT; T3 and T7 promoter sequences are in bold). PCR conditions were: 5 min 95°C; 5 cycles of 30 sec 95°C, 1 min 45°C, 1 min 72°C; 20 cycles of 30 sec 95°C, 1 min 65°C, 1 min 72°C and finally 5 min 72°C. The resulting PCR product was purified on a 1% agarose gel and sequenced. Aliquots of 200 ng were used for in vitro transcription labelling with biotin-16-UTP (Roche Diagnostics GmbH, Germany), using the MEGAscript-T3 (sense) or MEGAscript-T7 (anti-sense) kits, as described by the manufacturer (Ambion/ABI, Austin, TX, USA). To estimate quantity and labelling efficiencies, aliquots of the labelled RNA product were analysed by agarose gel (1%) electrophoresis. Bands from each primer combination were excised and sequenced for verification (DNA Technology, Aarhus, Denmark).

### In situ hybridisation

ISH was performed essentially as described previously [12]. The only deviation from the standard protocol was the removal of mercury from sections fixed in Stieve fixative [13] by 15 min treatment with iodide/potassium iodide (KI) (10 g/l)/I (5 g/l), followed by three washes in diethyl pyrocarbonate (DEPC) water. The iodine was subsequently removed by incubation in sodium thiosulphate pentahydrate (5% w/v, 10 min) followed by washing (3×DEPC water). The ISH procedure in brief: deparafinised sections were re-fixed in 4% parformadehyde (PFA), treated with proteinase K (P-2308; Sigma, USA) (1.0 or 2.5 μg/ml), post-fixed in PFA, pre-hybridized 1 h at 49°C, and hybridized overnight at 49°C with biotinylated antisense and sense control probes. Excess probe was removed with 0.1 × standard saline citrate (58°C) 3×30 min. Visualisation was performed using streptavidin conjugated with alkaline phosphatase (1:1000) (Cat. No. 1093266; Roche Diagnostics GmbH, Germany) followed by development with NBT BCIP.

## Results and Discussion

### Identification of the gonads in juvenile zebrafish

**Figure 1:**
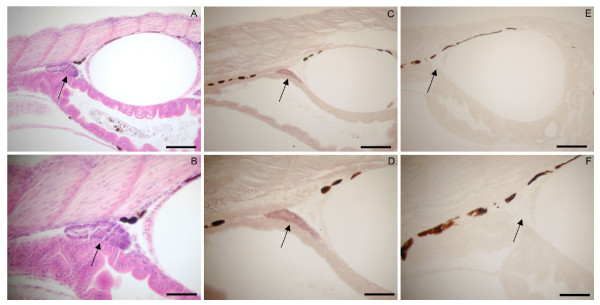
**Location of gonads in juvenile zebrafish (5 dph)**. A-B) HE staining C-D) ISH expression of *vasa *with antisense probe. E-F) ISH expression of *vasa *with sense probe. Upper panel is all 10×magnitude, scale bar: 100 μm. Lower panel 20×magnitude, scale bar: 50 μm.

Several techniques are commonly used to identify the gonads of juvenile zebrafish including HE staining of fixated zebrafish and *in situ *hybridisation (ISH) with a probe for a gonad specific gene. Previously, *vasa *has been used as a gonad specific gene in several fish species showing exclusive expression in germ cells of rainbow trout [14], medaka (the *vasa *orthologue *olvas*) [15] and zebrafish [16]. Furthermore, earlier studies have determined that *vasa *is expressed in the germ cells of both female and male zebrafish [16,17], making it a suitable gonadal marker in this species [17]. However, none of the above-mentioned techniques enables quantitative analysis of several gonadal genes during zebrafish sex differentiation. In this study, we have used both HE staining and ISH with *vasa *as probe to ensure that the NBT BCIP staining protocol is specific to the gonad. The location of the gonad visualised by HE staining (Figure 1A-B) corresponded with the location of the gonad detected by ISH with a *vasa *probe (Figure 1C-D). The strictly gonad-specific staining with *vasa *ensured that *vasa *could also serve as RT-PCR control gene for the specificity of the microdissected tissue.

### NBT BCIP staining for alkaline phosphatase

**Figure 2:**
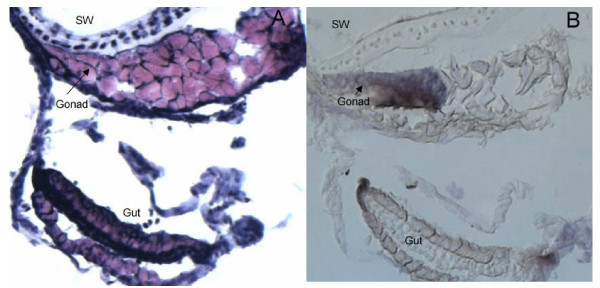
**Serial cryosections of a 10 dph juvenile zebrafish stained with A) HE and B) NBT BCIP**. The location of the swim bladder (SW), gonad and gut is shown.

**Figure 3:**
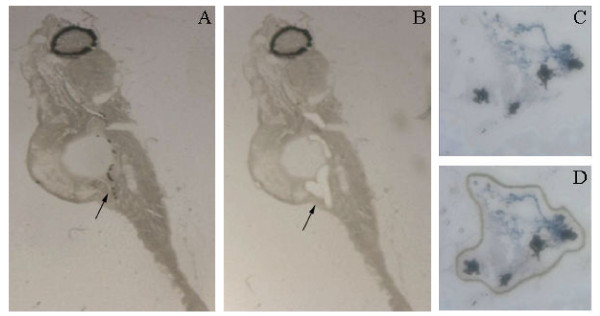
**Microdissection of gonads from 5 dph zebrafish**. A) Cryosection where gonads are stained with NBT BCIP before microdissection. B) Cryosections after microdissection of gonads. C) Gonad stained with NBT BCIP before microsdissection. D) Gonad stained with NBT BCIP after microdissection but before the marked section of tissue was removed from the membrane. Arrows indicate location of the gonad.

**Figure 4:**
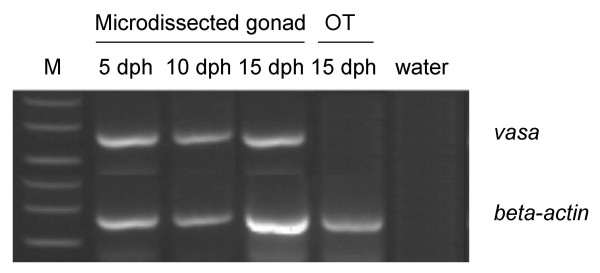
**RT-PCR expression of *vasa *and β-actin in microdissected tissue from juvenile zebrafish**. Microdissected gonad from: 5 dph, 10 dph and 15 dph zebrafish, microdissected other tissue than gonad (OT) from: 15 dph zebrafish and water: water control in PCR.

The morphology of cryo-sectioned dehydrated tissue is inferior compared to tissue in fixative and when membrane slides used for microdissection are used instead of glass slides, the morphology is further impaired. Therefore, it is difficult to distinguish between different organs in juvenile zebrafish and a specific staining method to the tissue of interest is absolutely necessary. In a previous study, Sonne et al. (2009) stained gonocytes in human testis for expression of alkaline phosphatase visualized with NBT BCIP and used the microdissected cells for microarray analysis [18]. As the expression of alkaline phosphatase is conserved in zebrafish gonocytes, we adapted the method to stain fetal germ cells in gonads of juvenile zebrafish. Staining of zebrafish cryosections with NBT BCIP showed a clear colouring of the gonad and HE staining of the next serial cryocut confirmed the location of the gonad (Figure 2). This ensured that when zebrafish cryosections are stained with NBT BCIP it is in fact the germ cells of the gonad that are specifically stained and this allows microdissection of cryosections with impaired morphology. The NBT BCIP staining was specific to the gonad, however, in all investigated fish we also observed staining of the eyes inside the natural black pigmentation (Figure 3A-B). The morphology of the NBT BCIP stained cryosections was compatible with laser microdissection and yielded RNA of a quality suitable for further analysis including RT-PCR. However, to obtain enough tissue for RNA purification it was necessary to microdissect serial cryocuts corresponding to the entire gonad of each individual fish (Figure 3C-D), which amounted to approximately 2 × 10^6 ^μm^2^. After RNA purification and two rounds of linear amplification a total amount of 21 -180 μg RNA was available from each gonad. To ensure that the microdissected tissue indeed was from the gonads, expression of *vasa *was investigated by RT-PCR. Figure 4 shows that *vasa *was expressed in the three samples of microdissected gonads whereas it was not detected in microdissected non-gonadal tissue, which primarily consisted of muscle tissue. To our knowledge, this is the first study allowing isolation of the gonad from individual juvenile zebrafish. However, a recent study by Vinas & Piferrer (2008) described laser microdissection of different spermatogenic stages from Mayer haematoxylin stained testis of adult sea bass males [19].

### RNA quality

**Figure 5:**
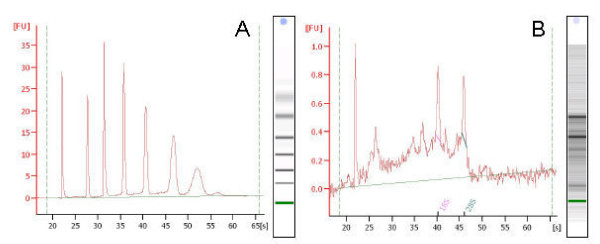
**BioAnalyzer pico gels**. A) Ladder and B) RNA purified from a microdissected gonad of a 15 dph zebrafish.

The best morphology and the highest RNA quality were obtained when the alkaline phosphatase staining protocol was applied to cryocuts fixed in 75% ethanol [11]. However, the staining itself resulted in a rapid decrease in RNA quality and in this study we performed the microdissection as fast as possible after staining in order to minimise RNA degradation. The quality of the purified RNA from microdissected gonad was analysed using BioAnalyzer picogel, which allows a visual inspection of RNA integrity and generates ribosomal RNA ratios, a RNA Integrity Number (RIN) that is a standardised RNA quality control (Figure 5). In this study the visual inspection of the picogel was weighted more important than the RIN value as the RIN application states that accurate values cannot be obtained below concentrations of 25 ng/μl. We measured concentrations of only 10-20 ng RNA/μl after the first round of linear application using a Nanodrop spectrophotometer, which means that the concentration in the sample before amplification is much lower most likely in the pg/μl range and therefore not within the limits that allow determination of accurate RIN values. Both the visual inspection of the picogel and the RIN values (3.5-5.5) indicated some degradation of RNA. However, according to earlier studies, it is possible to investigate gene expression profiles obtained from partially degraded RNA, which still have visible ribosomal bands and obtain reliable results if used carefully [3,9,10,12,20]. The linear amplification method used in this study has the advantage compared to exponential RNA amplification methods, such as reverse transcriptase-PCR, that it maintains the representation of the starting RNA population [6,21,22]. However, when comparing to the exponential PCR based approach the amplification is not as efficient. Generally, amplification of microdissected tissue that is partially degraded does inevitable result in RNA truncated corresponding to a loss of the 5'-end of the transcript [23] and therefore all primers used in this study lies within the 3'-end of the gene.

### Applicability to other species

The specific staining method to identify gonads of juvenile zebrafish described in this study takes advantage of the fact that germ cells have a high alkaline phosphatase activity, which can be detected by staining with NBT BCIP [8,11,24-26] Alkaline phosphatase activity appears to be conserved among vertebrate species and the staining protocol described in this study should therefore also be applicable to other vertebrate species. However, staining gonocytes to identify the gonad might only be applicable during a specific period in development when gonocytes are present. In zebrafish, the migration of primordial germ cells towards the developing gonads seems to be completed around 24 hours post fertilisation [27-29] and it should be possible to stain and microdissect zebrafish gonads from this time in development. We have successfully stained and microdissected gonads from 2 dph zebrafish. However, as the gonocytes differentiate to spermatogonia or oocytes they might loose expression of alkaline phosphatase activity and therefore the gonads may not be detectable with the NBT BCIP staining method in adult zebrafish. For example, murine PGCs *in vivo *loose their alkaline phosphatase activity around 14.5 dpc [30]. In this study, we have successfully stained and microdissected gonads from individuals that were up to 20 dph but whether the staining method is applicable to older fish have not been investigated.

In conclusion, we have established a protocol that enables laser microdissection of gonads from serial cryosections of juvenile zebrafish, allowing subsequent RNA purification and amplification. Expression of the conserved germ cell-specific gene *vasa *was investigated by ISH to ensure that *vasa *was specifically expressed in the gonads and then by RT-PCR to ensure that the stained and microdissected tissue was indeed gonadal. Future investigations should include determination of gonadal gene expression of genes that have previously been implicated in zebrafish sex determination and differentiation.

## Competing interests

The authors declare that they have no competing interests.

## Authors' contributions

AJ conducted the NBT BCIP staining, microdissection, RNA purification, RNA amplification and RT-PCR. AJ and JEN conducted the ISH experiment. JEM setup the zebrafish and collected the fish. JEN, PB and HL participated in the planning of experiments, devised the study and participated in the discussion of results. AJ and HL wrote the manuscript. All authors read, commented and approved the final manuscript.
